# Investigation on Ciliary Functionality of Different Airway Epithelial Cell Lines in Three-Dimensional Cell Culture

**DOI:** 10.1089/ten.tea.2019.0188

**Published:** 2020-04-16

**Authors:** Nina Lodes, Katharina Seidensticker, Alexander Perniss, Sarah Nietzer, Heike Oberwinkler, Tobias May, Thorsten Walles, Helge Hebestreit, Stephan Hackenberg, Maria Steinke

**Affiliations:** ^1^ Chair of Tissue Engineering and Regenerative Medicine, University Hospital Würzburg, Würzburg, Germany.; ^2^ Department of Oto-Rhino-Laryngology, Plastic, Aesthetic and Reconstructive Head and Neck Surgery, University Hospital Würzburg, Würzburg, Germany.; ^3^ Institute for Anatomy and Cell Biology, German Center for Lung Research DZL, Justus-Liebig-University Giessen, Giessen, Germany.; ^4^ Translational Center Regenerative Therapies, Fraunhofer Institute for Silicate Research ISC, Würzburg, Germany.; ^5^ Inscreenex GmbH, Braunschweig, Germany.; ^6^ Department of Thoracic Surgery, University Medicine Magdeburg, Magdeburg, Germany.; ^7^ Department of Pediatrics, University Hospital Würzburg, Würzburg, Germany.

**Keywords:** respiratory tissue models, ciliary beating, 3D cell culture

## Abstract

Three-dimensional respiratory tissue models have been generated using, for example, human primary airway epithelial cells (hAEC) or respective cell lines. To investigate ciliopathies, such as primary ciliary dyskinesia, the presence of functional kinocilia *in vitro* is an essential prerequisite. Since access to hAEC of healthy donors is limited, we aimed to identify a respiratory epithelial cell line that is capable to display functional kinocilia on at least 60% of the apical surface. Thus, we cultured four different human respiratory cell lines with human primary airway fibroblasts under airlift conditions, characterized the morphology, and analyzed ciliary function. Only one of the tested cell lines showed beating kinocilia; however, <10% of the whole surface was covered and ciliary beating was undirected. Positive control tissue models using hAEC and fibroblasts displayed expected directed ciliary beating pattern around 11 Hz. Our data show that the available cell lines are not suitable for basic and applied research questions whenever functional kinocilia are required and that, rather, hAEC- or human induced pluripotent stem cell-derived tissue models need to be generated.

**Impact Statement:**

To study ciliopathies or *Bordetella pertussis* infection *in vitro*, three-dimensional respiratory tissue models with functional kinocilia covering at least 60% of the model's surface are mandatory. We cultured four respiratory cell lines on a fibroblast-loaded biological scaffold and showed that none of them met this requirement. In contrast, primary airway cell-derived models sufficiently reflected the mucociliary phenotype. To further search for an alternative to primary respiratory cells, investigations on other cell lines should be conducted or even new cell lines have to be generated.

## Introduction

Three-dimensional (3D) cell cultures of the respiratory epithelium/mucosa afford research on—for example—the impact of airborne pollutants, host-pathogen interactions, and drug permeation.^1–5^ As source for respiratory epithelial cells, primary cells, induced pluripotent stem cells (iPSC), or cell lines can be used. Both primary cells and iPSC provide the opportunity to generate personalized tissue models, for example, to study individual drug responses or drug efficacy. Moreover, they show a high *in vitro-in vivo* correlation. These models feature a pseudostratified epithelial morphology, barrier properties, basal cells, mucus-producing goblet cells, and ciliated cells facilitating mucociliary clearance.^6–9^ However, primary cell cultures are difficult to standardize and to establish in large quantities due to shortness of donor cells and donor variability. Moreover, because of their low passaging capability,^10^ primary respiratory epithelial cells are rather unsuitable to be used for gene editing. In contrast, cell lines show greatly enhanced life span and are standardizable. Depending on the airway epithelial cell line used, the 3D tissue models show distinct features of the mucociliary phenotype, such as epithelial cell polarization, mucus production, or barrier integrity.

However, the presence of functional kinocilia in such tissue models appears to be a great challenge. Some studies have already documented ciliated cells in cell line-based 3D respiratory tissue models. For example, it was reported that kinocilia of the VA10 cell line covered up to 15% of the tissue model's surface area, exhibiting a beating frequency of 6–7 Hz when seeded on transwell inserts and cultured under air-lift conditions.^1^ The cell line HBEC3-KT that was seeded on fibroblast-loaded collagen gels developed kinocilia; however, there is only little information on ciliary functionality.^11^

To investigate distinct research topics using 3D respiratory epithelial/mucosal tissue models, such as host-pathogen interaction of the respiratory epithelium with *Bordetella pertussis* that requires the presence of kinocilia for adherence^12^ or ciliopathies, for example, primary ciliary dyskinesia (PCD),^13^ functional kinocilia and, thus, mucociliary transport are mandatory. The aim of this study was to identify human airway epithelial cell lines that can be used to generate 3D respiratory tissue models comprising the mucociliary phenotype. At least 60% of the apical surface should be covered with kinocilia that show a directed beating pattern to make it comparable with the situation *in vivo*, where 60–80% of the epithelium consists of ciliated cells.^14^

Since it is known from previous work that the composition of 3D scaffolds and/or the co-culture with other cell types can impact the phenotype of airway epithelial cells *in vitro*,^15–17^ we used a porcine small intestinal submucosa (SIS) scaffold loaded with airway tissue-derived fibroblasts and compared our data to transwell-based assays.

## Materials and Methods

### Donor tissue and primary cells

Human respiratory mucosa was recovered from tracheobronchial surgical specimens that were obtained in anatomical lung resections (three male donors, 28–68 years old). For fibroblast isolation, specimens from human nasal mucosa of two male donors (33 and 55 years old) were obtained during surgery on the nasal airways in the Department of Otorhinolaryngology, Plastic, Aesthetic and Reconstructive Head and Neck Surgery of the University Hospital Würzburg. The tissue was exclusively taken from the uncinate process being a structure that is removed during sinus surgery in most cases. In contrast to the inferior turbinate, which is often used as a source of mucosa biopsy, the uncinate process is covered by the respiratory epithelium without metaplastic regions.

Patient's informed consent was obtained before surgery and the studies were approved by the institutional ethics committees on human research of the Otto-von-Guericke University Magdeburg (vote 163/17) and Julius-Maximillians-University Würzburg (vote 182/10 and 179/17), respectively.

### Two-dimensional cell culture

For generation of the CI-huAEC cell line, primary human bronchial epithelial cells were obtained from Promocell GmbH. The CI-huAEC cell line was established by transduction of a gene library composed of 33 different genes^18^ and has the following genes integrated: *c-myc*, *Id3*, *Bcl2*, *HCV core protein*, *Nanog*, *Lmo2*, and *Rex1*. The human airway epithelial cell lines Cl-huAEC (Inscreenex GmbH, Braunschweig, Germany) and Calu-3 (ATCC^®^ HTB-55^™^, Manassas, VA) were handled according to provider's instructions and cultured in huAEC (INS-ME-1013-500ml; Inscreenex) and minimum essential medium (No. 11095080; Thermo Fisher Scientific, Waltham, MA) supplemented with 10% fetal calf serum (FCS) and 1% sodium pyruvate, respectively.

VA10 cells were kindly provided by Prof. G.H. Gudmundsson (Biomedical Center and Department of Life and Environmental Sciences, University of Iceland, Reykjavík, Iceland) and cultured in Airway Epithelial Cell Growth (AECG) Medium (No. PB-MH-350-0099; Pelo Biotech). HBEC3-KT cells (ATCC CRL-4051^™^) were grown in Keratinocyte-SFM (K-SFM, No. 17005042; Thermo Fisher Scientific) according to a previously published protocol.^19^ Human primary airway epithelial cells (hAEC) and human airway fibroblasts were isolated and cultured in AECG medium and Dulbecco's modified Eagle's medium (DMEM) supplemented with 10% FCS, respectively, as described elsewhere.^7^ However, in this study, antibiotics were omitted in fibroblast cultures. All the cells were cultured under standard conditions (37°C and 5% CO_2_) and medium was replaced three times per week.

### 3D human airway tissue models

3D airway mucosa tissue models were generated based on decellularized porcine SIS according to a previously published protocol.^7^ We used 250,000 airway epithelial cells (hAEC, HBEC3-KT, VA10, Calu-3, or Cl-huAEC) per cm^2^ and 50,000/cm^2^ fibroblasts derived from human airway mucosal tissue. We aimed to assess a possible impact of the 3D scaffold (SIS vs. transwell inserts) on the epithelial morphology of HBEC3-KT. Thus, we cultured 44,000/cm^2^ primary airway fibroblasts and 220,000/cm^2^ HBEC3-KT on transwell inserts (0.4 μm, translucent; Greiner Bio-One). MucilAir^™^ tissue models (primary bronchial hAEC and fibroblasts on transwell inserts from Epithelix Sàrl, Geneva, Switzerland, one female donor and two male donors, 15–66 years old) served as positive controls and were cultured according to the manufacturer's instructions.

All 3D cultures based on epithelial cell lines were cultured for 21 days and the tissue models based on primary epithelial cells (MucilAir™ and hAEC on SIS) were cultured for 30 days. The tissue models were cultured under airlift conditions and cell culture medium was changed three times per week. The culture media were composed of 50% fibroblast culture medium and 50% of the respective airway epithelial cell medium.

### Histological and immunofluorescent staining

After fixation of the 3D airway tissue models with 4% Roti^®^-Histofix, they were embedded in paraffin and cross-sectioned at 3 μm thickness. Deparaffination and rehydration of the sections were performed in xylene and a descending ethanol series. Hematoxylin and eosin (H&E) staining was performed according to standard protocols. For immunofluorescent staining, the following primary antibodies were used: monoclonal mouse anti-cytokeratin 5/6 (CK5/6, 1:100, M7237; Dako, Eching, Germany), monoclonal rabbit anti-Cytokeratin 18 (CK18, 1:1000, NBP2-67370; Novus Biologicals, Littleton, CO), monoclonal rabbit anti-vimentin (VIM, 1:3000, ab92547; Abcam, Cambridge, United Kingdom), monoclonal mouse anti-Mucin 5AC (MUC5AC, 1:1000, MA5-12178; Thermo Fisher Scientific), polyclonal rabbit anti-Mucin 5B (MUC5B, 1:100, HPA008246; Sigma-Aldrich, Munich, Germany), and monoclonal mouse anti-β tubulin (β-Tub, 1:2000, T4026; Sigma-Aldrich). Primary antibodies MUC5AC, MUC5B, VIM, and β-Tub required antigen retrieval at pH 6, whereas CK5 and CK18 required antigen retrieval at pH 9.

Primary antibodies were incubated overnight at 4°C and secondary antibodies were incubated at room temperature for 1 h. For primary antibody detection, polyclonal donkey anti-rabbit antibodies coupled to Alexa Fluor 488 (1:400, A-21206; Thermo Fisher Scientific) and polyclonal donkey anti-mouse antibodies coupled to Alexa Fluor 555 (1:400, A-31570; Thermo Fisher Scientific) were used, respectively. Sections were mounted with DAPI Fluoromount-G^®^. Images were taken with the BZ-9000 BIOREVO System (Keyence, Neu-Isenburg, Germany). To characterize all 3D tissue models based on the SIS, four independent experiments were performed, each. With HBEC3-KT on transwell inserts and MucilAir™, four and three independent experiments were performed, respectively.

### Sample preparation for ultrastructural analysis

Ultrastructural analysis was carried out at the Imaging Core Facility of Prof. Dr. Christian Stigloher (Division of Electron Microscopy, Biocenter of the University of Würzburg) according to a previously published protocol.^20^ In brief, samples were fixed in 2.5% glutaraldehyde, then fixed in 2% OsO_4_, washed with H_2_O, and incubated in 0.5% uranyl acetate. Then, the samples were dehydrated, embedded in Epon812, ultrathin sectioned, and subsequently analyzed with a Zeiss EM10 (Zeiss/LEO, Oberkochen, Germany). With HBEC3-KT, Calu-3, and Cl-huAEC, three independent experiments and with VA10 one experiment was performed.

### Measurement of ciliary beating frequency and statistical analysis

For functional analysis of kinocilia, 3D tissue models were removed from the inserts and placed on delta T-dishes, which were positioned in a delta T culture dish controller plate (Bioptechs, Butler, PA). This setup served as a microenvironmental control system enabling constant temperature (37°C) during the measurements as ciliary beating frequency (CBF) is temperature sensitive.^21^ The dishes were coated with a polymer beforehand (Sylgard; Dow Corning, Wiesbaden, Germany), allowing the tissue models to be fixed with small pins. The airway tissue models were submerged in the cell type-specific culture medium. Videos were taken using a water immersion objective (Fluor 20 × /0.50 W and NIR Apo 40 × /0.80 W; Nikon, Tokyo, Japan) and a high speed camera (Genie HM640; Teledyne Dalsa, Waterloo, Canada).

Only tissue models using HBEC3-KT, hAEC on SIS, and MucilAir™ were analyzed. From the apical surface of each 3D tissue model, one video was recorded (1–7 technical replicates, *n* = 3). Videos consisted of 1000 images (640 × 480 pixels) with a frame rate of 100 images/s and were taken every 10 min (30 min in total). In HBEC3-KT models based on the SIS, we recorded only one video of one technical replicate. Then, 10 regions of interest (ROIs) of each video of primary cell tissue models and of 3 ROIs of the video of the HBEC3-KT were analyzed. Student's *t*-test (two-sided) was performed with the arithmetic means of ROI of 1–7 technical replicates out of three single experiments (*n* = 3). For *p* < 0.05, differences were defined as being significant. All statistical analysis was done with Microsoft Excel 2013. CBF was determined by fast Fourier transformation using a graphical user interface for MATLAB, which was programmed by Prof. Dr. Peter König (University of Lübeck, Lübeck, Germany).^22^

## Results

**FIG. 1.**
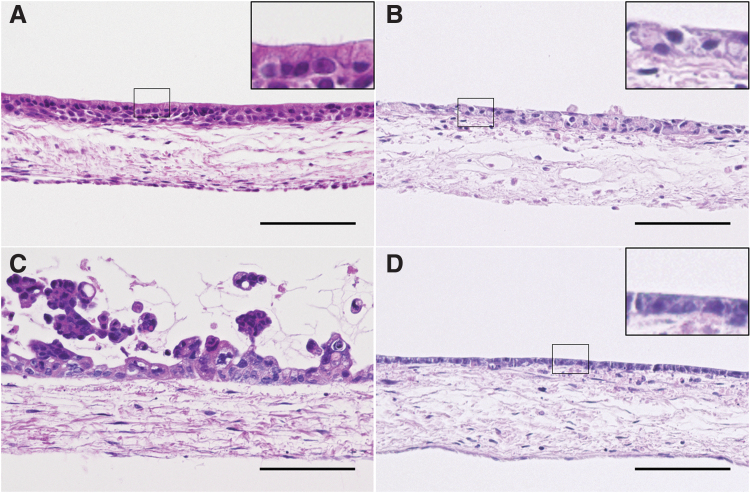
H&E staining of 3D airway mucosa tissue models using different respiratory epithelial cell lines. 3D cell culture of HBEC3-KT-based models reveals a polarized epithelial cell layer **(A)** containing kinocilia-like structures on the apical surface **(A**, *inset***)**. VA10-based models show a rather undifferentiated epithelial layer **(B)**. Calu-3-based models show cell cluster formation **(C)**. Cl-huAEC-based models show a polarized cell monolayer on the apical surface **(D)**. Calu-3-, VA10-, and Cl-huAEC-based models do not show kinocilia-like structures **(B–D)**. Scale bars: 100 μm. 3D, three dimensional; H&E, hematoxylin and eosin.

Histological analysis of H&E-stained 3D tissue models, which were generated using the different airway epithelial cell lines and fibroblasts on the SIS, revealed diverse, epithelial cell type-specific morphology. HBEC3-KT-based tissue models showed a polarized epithelium and kinocilia-like structures on the apical side (Fig. 1A). VA10 cultures appeared similar; however, no kinocilia-like structures were found (Fig. 1B). Calu-3 cells formed multilayered cell clusters on the apical side of the SIS (Fig. 1C). Cl-huAEC polarized and built a consistent monolayer (Fig. 1D).

**FIG. 2.**
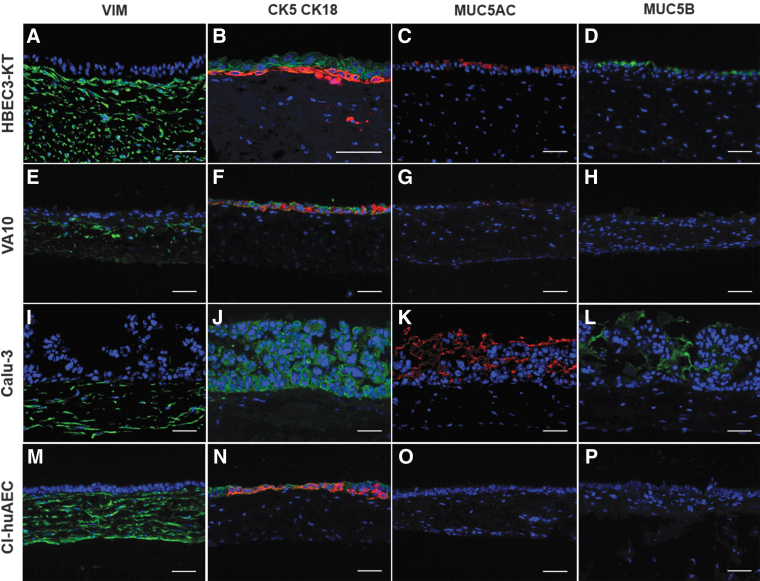
Immunofluorescent staining of vimentin, CK5, CK18, MUC5AC, and MUC5B in 3D airway mucosa tissue models. Vimentin is localized in the SIS of each cell line-based model **(A, E, I, M)**. 3D tissue models based on HBEC3-KT, VA10, and Cl-huAEC show CK5 (*red*) and CK18 (*green*) expression in the epithelial compartment **(B, F, N)**, whereas Calu-3-based models show only CK18 expression **(J)**. MUC5AC and MUC5B are identified in HBEC3-KT- **(C, D)**, VA10- **(G, H),** and Calu-3-based models **(K, L),** but are not visible in Cl-huAEC-based models **(O, P)**. Scale bars: 50 μm. CK, cytokeratin; MUC, mucin; SIS, small intestinal submucosa.

Immunofluorescent staining was carried out to determine airway mucosa-specific cell types and mucins. VIM-positive fibroblasts that migrated into the SIS were verified in all cell line-based airway mucosa tissue models on the SIS (Fig. 2A, E, I, M). In 3D tissue models based on HBEC3-KT, VA10, and Cl-huAEC, both CK5- and CK18-specific signals were found in the epithelial compartment (Fig. 2B, F, N).

Calu-3-based tissue models showed only CK18-specific staining (Fig. 2J). Whereas in HBEC3-KT and Cl-huAEC models, CK5-positive cells were mostly found adjacent to the SIS and CK18-positive cells polarized toward the apical side; in VA10 models, CK5- and CK18-positive cell arrangement was unorganized. The two most important mucins of the conducting airways, MUC5AC and MUC5B, were identified in HBEC3-KT- and Calu-3-based models on the epithelial surface (Fig. 2C, D, K, L). In VA10-based models, both mucins were present; however, the fluorescent signals were qualitatively lower (Fig. 2G, H). The Cl-huAEC-based tissue models did not display either MUC5AC- or MUC5B-specific signals (Fig. 2O, P and Table 1).

**Table 1.** Qualitative Overview of the Mucociliary Phenotype of Three-Dimensional Airway Mucosa Tissue Models Used in This Study

	SIS					Transwells	
	HBEC3-KT	VA10	Calu-3	Cl-huAEC	hAEC	HBEC3-KT	MucilAir^™^
Basal cells (CK5)	+	+	−	+	+	+	+
Mucins (MUC5AC/5B)	++/++	+/+	+++/+++	−/−	+/+	++/++	+/+
Differentiated epithelial cells (CK18)	+	+	+	+	+	+	+
Ciliary beating	−/+	−	−	−	+	−	+

CK, cytokeratin; hAEC, human primary airway epithelial cells; SIS, small intestinal submucosa.

**FIG. 3.**
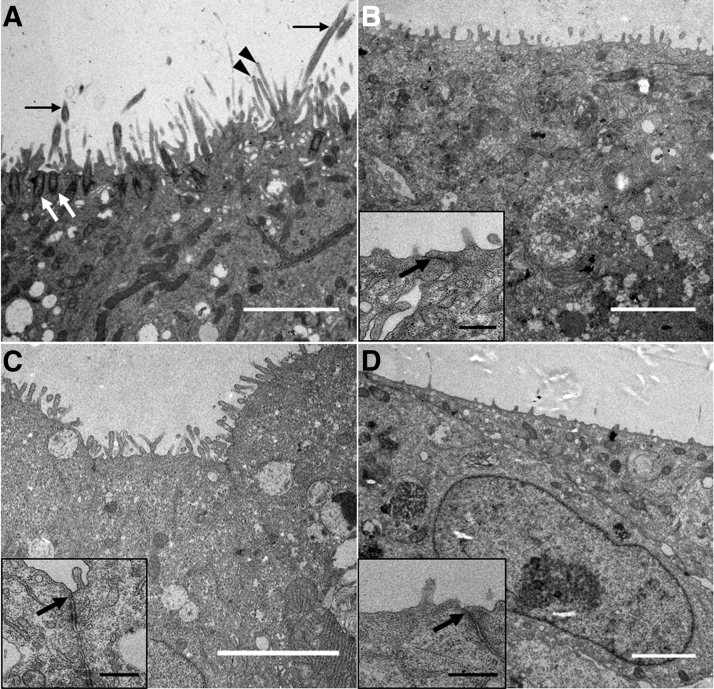
Ultrastructure of 3D airway mucosa tissue models using different airway epithelial cell lines. HBEC3-KT-based models show kinocilia on the apical surface (*black arrows*). Basal bodies are clearly visible (*white arrows*) and microvilli (*black arrowheads*) line the epithelial surface **(A)**. Tissue models generated with VA10 **(B)**, Calu-3 **(C)** or Cl-huAEC **(D)** show microvilli and tight junctions **(***arrows* in *insets* **B**–**D**, respectively**)** on the epithelial cell surface. Scale bars: 3 μm **(A–D)**, 0.5 μm (*insets*).

To investigate the epithelial surface of the different cell line-based 3D airway mucosa tissue models in detail, ultrastructural analyses were performed. In HBEC3-KT-based models, we identified kinocilia with clearly visible basal bodies and microvilli on the epithelial surface (Fig. 3A). The models generated using VA10, Calu-3, or Cl-huAEC showed microvilli on the apical side, too, but lacked kinocilia (Fig. 3B–D). Moreover, VA10, Calu-3, and Cl-huAEC built tight junctions (insets in Fig. 3B–D).

**FIG. 4.**
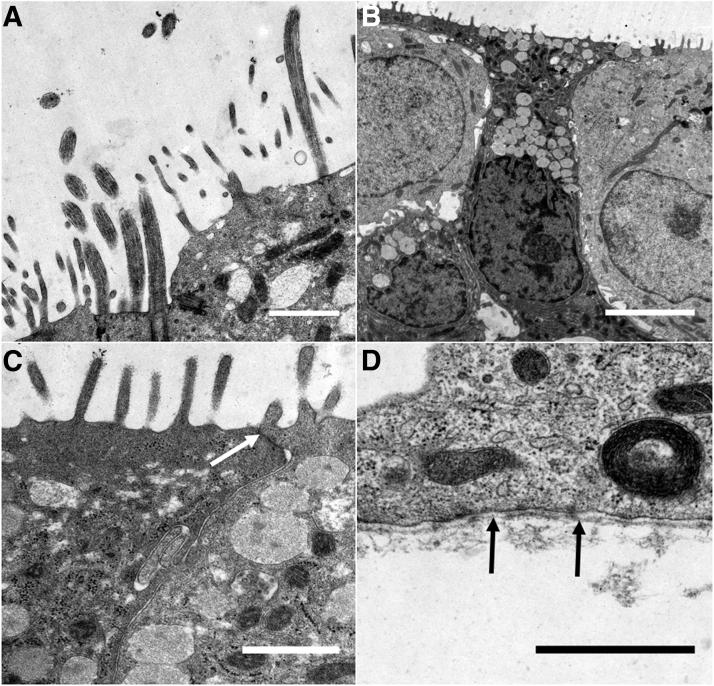
Ultrastructural analysis of HBEC3-KT-based airway mucosa tissue models. Ciliated cells **(A)** and mucus-producing goblet cells **(B)** can be observed in 3D cell culture. Tight junctions **(C**, *arrow***)** and a basement membrane **(D**, *arrows***)** are also visible. Scale bars: 1 μm **(A, C, D)** and 5 μm **(B)**.

Since HBEC3-KT were the only cells that differentiated into a ciliated phenotype when cultured on the SIS, these models were assessed in further ultrastructural analysis. Besides ciliated epithelial cells (Fig. 4A), we identified mucus-producing goblet cells (Fig. 4B) and tight junctions (Fig. 4C). Moreover, the epithelial layer was anchored by a basement membrane to the underlying connective tissue (Fig. 4D).

Tissue models using VA10, Calu-3, and Cl-huAEC on SIS did not display any kinocilia and HBEC3-KT on transwells showed only immotile kinocilia. Thus, CBF analysis was not performed. Due to the low quantity of beating kinocilia in HBEC3-KT on SIS, ciliary beating of those could be analyzed for only one technical replicate. The beating pattern was undirected and CBF was 6.9 ± 0.1 Hz (Fig. 6C and Supplementary Video S1).

**FIG. 5.**
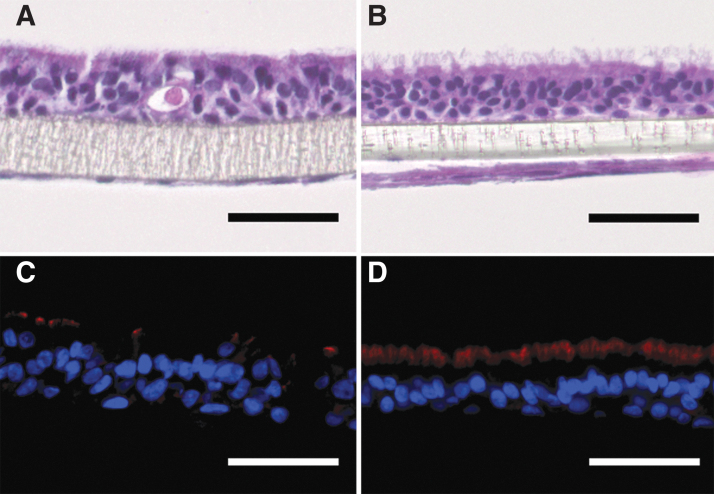
Morphological characterization of airway mucosa models on transwell inserts using primary airway fibroblasts and HBEC3-KT **(A, C)** or hAEC **(**MucilAir™; **B, D)**. H&E stainings show differentiated epithelial cell layers **(A, B)** with beta tubulin-positive kinocilia **(***red fluorescence* in **C, D)**. Scale bars: 50 μm. hAEC, human primary airway epithelial cells.

To investigate a possible impact of the 3D scaffold on the differentiation of HBEC3-KT, we compared the morphology of HBEC3-KT and fibroblasts on transwell inserts to the respective SIS-based 3D tissue models. When seeded on transwell inserts, HBEC3-KT showed a polarized epithelial morphology with kinocilia-like structures on the apical surface, which was comparable to 3D tissue models based on SIS (Figs. 1A and 5A). Immunofluorescent staining against β-Tub verified kinocilia on the apical surface of HBEC3-KT on transwells (Fig. 5C). However, only immotile kinocilia were found and MucilAir™ showed the typical mucociliary phenotype (Fig. 5B) with β-Tub-positive kinocilia (Fig. 5D). Compared to HBEC3-KT on transwell inserts, MucilAir™ displayed more β-Tub-positive kinocilia with directed beating (Supplementary Video S2). Both MucilAir™ and HBEC3-KT on transwells showed the typical marker in immunofluorescent stainings (Supplementary Fig. S1). hAEC on the SIS differentiated well as published elsewhere^6,7^ and showed directed ciliary beating (Supplementary Video S3).

**FIG. 6.**
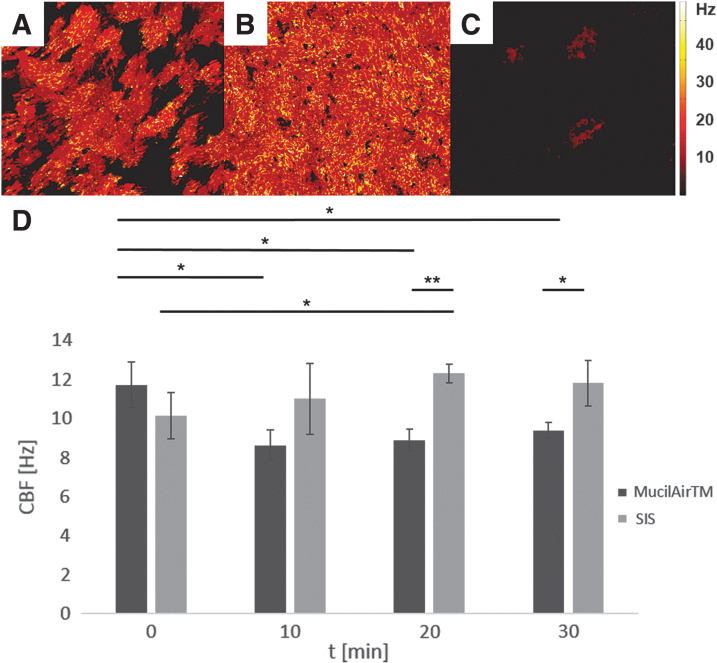
Heat maps show the distribution of detected CBF of hAEC on the SIS **(A)**, MucilAir^™^ **(B),** and HBEC3-KT on the SIS **(C)**. CBF of primary airway tissue models based on either transwell inserts (MucilAir™) or a decellularized porcine SIS over 30 min **(D)**. Data are presented as arithmetic means (bars) ± SD (error bars), *n* = 3, student's *t*-test, two sided, *p < 0.05, **p < 0.005. CBF, ciliary beating frequency; SD, standard deviation.

MucilAir™ and hAEC on the SIS showed beating kinocilia that covered at least 60% of the apical surface, as seen in respective heat maps (Fig. 6A, B). Only with these tissue models, CBF analysis with subsequent statistical testing could be done. MucilAir™ showed a significant decrease from 11.7 ± 1.2 to 8.6 ± 0.8 Hz, 8.9 ± 0.6 Hz, and 9.4 ± 0.4 Hz, in CBF after 10, 20, and 30 min, respectively. CBF of SIS-based tissue models significantly increased after 20 min from 10.1 ± 1.2 to 12.3 ± 0.5 Hz and remained constant at 11.3 ± 0.9 Hz after 30 min. Comparing MucilAir™ and SIS-based tissue models, CBF in SIS-based models was significantly higher after 20 and 30 min (12.3 ± 0.5 Hz vs. 8.9 ± 0.6 Hz and 11.8 ± 1.2 Hz vs. 9.4 ± 0.4 Hz) (Fig. 6D).

## Discussion

In this study, we aimed to identify an airway epithelial cell line that was capable to differentiate to the mucociliary phenotype. Special attention was payed to assess the presence of functional kinocilia on at least 60% of the tissue model´s surface that is important, for example, for PCD or *B. pertussis* research. On the fibroblast-loaded biological scaffold that we used (SIS), only HBEC3-KT cells differentiated to the mucociliary phenotype, whereas Calu-3, VA10, and Cl-huAEC showed only partial features of respiratory epithelium and no kinocilia.

Calu-3 formed multilayered cell clusters on the apical surface of the scaffold, were partly polarized, and showed MUC5AC, MUC5B, microvilli, and tight junctions. Except for the presence of cell cluster, Calu-3 showed similar morphological characteristics compared to previous studies that were performed on transwell inserts.^23–25^ To our knowledge, there is no study that could verify kinocilia on Calu-3 cells at the air-liquid interface. To verify basal cells in the tissue models, we performed CK5 immunofluorescent staining. Calu-3 were CK5-negative, meaning that this cell line did not feature precursor-like cells. It has been shown that VA10 are able to differentiate into ciliated cells with a CBF of 6 to 7 Hz when cultured on transwell membranes. The ciliated cells covered 10 to 15% of the tissue models' surface.^1^ We investigated if the chosen 3D scaffold and addition of primary human airway fibroblasts could improve the mucociliary phenotype. However, when seeded on the SIS, VA10 lacked kinocilia, built an epithelial layer containing microvilli and tight junctions, CK5-positive basal, and CK18-positive differentiated cells, and showed a qualitatively low amount of the mucins 5AC and 5B. The arrangement of basal and differentiated epithelial cells was unorganized. Thus, our culture conditions could not enhance the ciliary phenotype of VA10-based tissue models.

3D airway mucosa tissue models using Cl-huAEC showed a polarized epithelial layer with tight junctions, microvilli on the surface, and partly well-arranged CK5- positive basal and CK-18-positive differentiated epithelial cells. However, neither kinocilia nor the mucins 5AC and 5B could be detected on the epithelial surface. Thus, when cultured on the fibroblast-loaded SIS, Cl-huAEC mimics only few features of the human upper respiratory epithelium. Further analyses will need to clarify if this cell line rather represents the lower respiratory tract investigating respective markers, such as surfactant proteins.

When cultured on the SIS, HBEC3-KT tissue models showed the closest morphological similarity to the mucociliary phenotype *in vivo*. However, undirected beating kinocilia covered <5% of the model's surface, which makes it unsuitable for studies where high numbers of functional kinocilia are required. When cultured on transwell inserts, we detected only immotile kinocilia. Others reported kinocilia on the apical surface of 3D tissue models on transwell inserts, Matrigel, or collagen gels using HBEC3-KT.^11,19,26^ However, only little information on cilia functionality and surface coverage has been available. The SIS shows a connective tissue-like architecture made of mostly collagen and elastin. Moreover, SIS contains glycoproteins, glycosaminoglycans, proteoglycans, and distinct growth factors. These biomechanical and biochemical properties facilitate cell attachment, adhesion, proliferation, and differentiation,^27^ and we assume that this environment favors the higher grade of differentiation of HBEC3-KT on SIS compared to transwell inserts. In the 3D tissue models, the culture media were composed of 50% fibroblast culture medium and 50% of the respective airway epithelial cell medium, which could impact both fibroblasts and epithelial cells, for example, regarding growth factor secretion and differentiation. Subsequent studies could investigate possible effects of changing the media composition on the mucociliary phenotype.

Thus, culture conditions, such as the 3D scaffold, media, co-culture with other cells, and culture period, can all impact epithelial cell differentiation. Our data show that at least on the SIS and under defined, comparable culture conditions, the selected cell lines did not sufficiently differentiate to the mucociliary phenotype. Also, other groups reported on cell lines that did not display kinocilia when co-cultured with fibroblasts in 3D conditions.^28^ This implies that these cells should not be recruited for gene-editing experiments, for example, to model ciliopathies, such as PCD.

To verify that the generation of 3D respiratory tissue models on fibroblast-loaded SIS and transwell inserts is possible with our protocols, we cultured hAEC on the SIS and chose MucilAir™ as positive controls. Both tissue models showed directed ciliary beating and a stable CBF after 20 min around 8–13 Hz. Both model systems showed functional ciliary activity and were within the normal ciliary beating range of the upper respiratory tract. Previous *ex vivo* and *in vitro* studies using primary conducting airway epithelial cells showed beating frequencies between 4 and 19 Hz.^28–30^ The significant differences between MucilAir™ and hAEC in CBF observed in this study could be due to the anatomical region where the primary cells were obtained.^31^

To our knowledge, there are no published studies showing cell line-based 3D tissue models of human respiratory epithelium with functional kinocilia covering at least 60% of the apical surface. For research questions where a high number of functional kinocilia is mandatory, primary cells or hiPSC-derived cells should rather be used that enable adequate differentiation with a high *in vitro-in vivo* correlation of the model.

## Supplementary Material

Supplemental data

Supplemental data

Supplemental data

Supplemental data
