# Supplementary material for: Investigation on Ciliary Functionality of Different Airway Epithelial Cell Lines in Three-Dimensional Cell Culture
Source: Tissue Eng Part A. 2020 Apr 16;26(7-8):432–40. doi: 10.1089/ten.tea.2019.0188 (PMC7187987; doi:10.1089/ten.tea.2019.0188)
Supplement: Supplemental data [file Supp_FigS1.pdf]

Supplementary Data

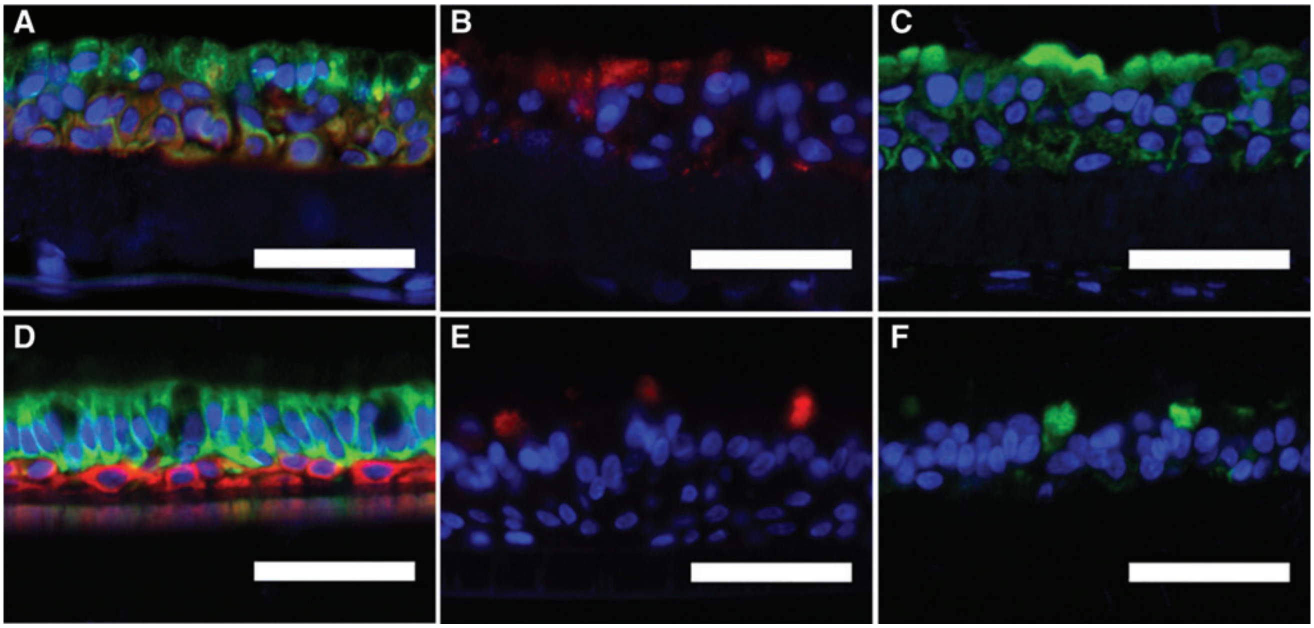

SUPPLEMENTARY FIG. S1. Immunofluorescent staining of fibroblasts and HBEC3-KT on transwell inserts **(A–C)** and MucilAir™ **(D–F)**. Both tissue models reveal CK5 (*red*)- and CK18 (*green*)-positive cells **(A, D)** and show MUC5AC **(B, E)** and MUC5B **(C, F)**. Scale bars: 50 µm. CK, cytokeratin; MUC, mucin.
